# Supplementary figures and images for: Genome-scale metabolic modeling reveals metabolic trade-offs associated with lipid production in Rhodotorula toruloides
Source: PLoS Comput Biol. 2023 Apr 26;19(4):e1011009. doi: 10.1371/journal.pcbi.1011009 (PMC10204961; doi:10.1371/journal.pcbi.1011009)

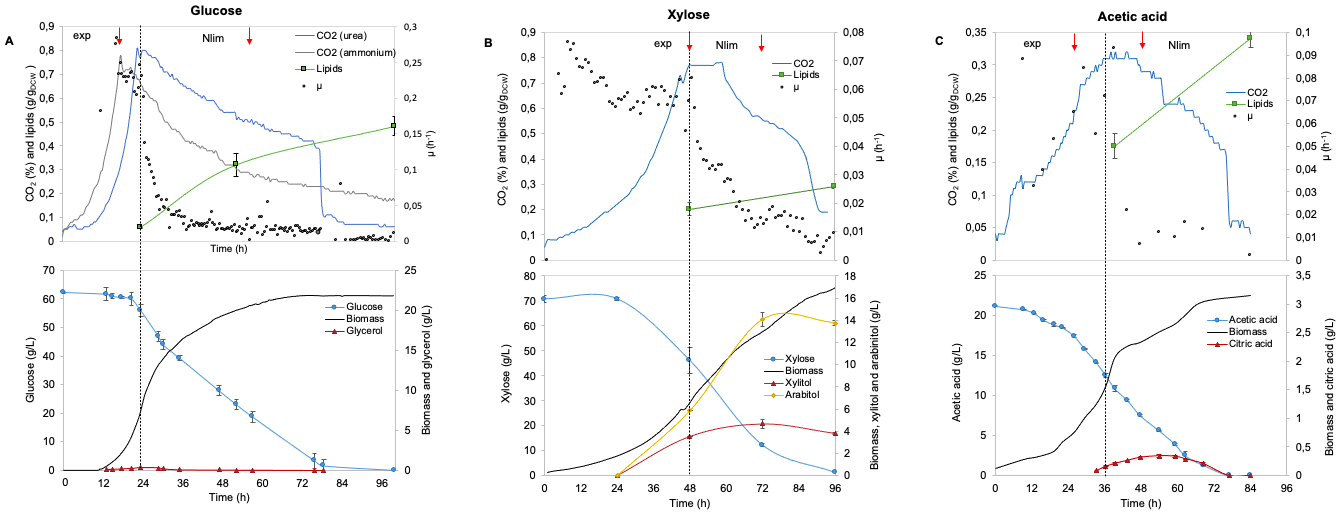

Supplement: S1 Fig — (A) glucose (63 g/L, C/N 68.6), (B) xylose (70g/L, C/N 80) and (C) acetate (20 g/L, C/N 80). Arrows in red are used to denote sampling points for proteomics and protein content measurements. Average of duplicate experiments with SD in extracellular metabolites concentration (g/L) and intracellular lipid content (g_lipid/gDCW) is illustrated. Curves represent a single measurement in bioreactor 2 (R2) in CO2 (%), specific growth rate μ (h-1) and biomass concentration (g/L), while for the rate calculations used for modelling duplicate conditions were used. (TIF) [file pcbi.1011009.s014.tif]

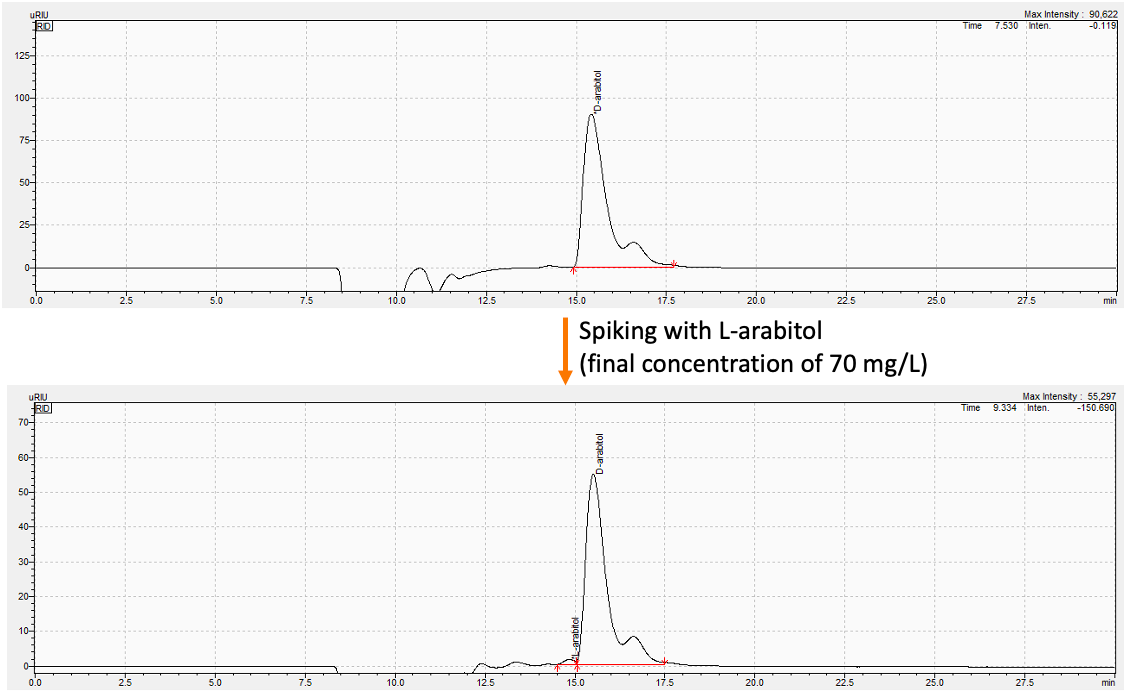

Supplement: S2 Fig — Figures represent HPLC profiles of D-arabinitol during nitrogen limitation phase on xylose (XNlim) performed at 20°C. Column: Chiralpak; eluent: hexane-ethanol (70,30, v/v). Flow rate 0.3 mL/min; detection: refractive index. (TIF) [file pcbi.1011009.s015.tif]

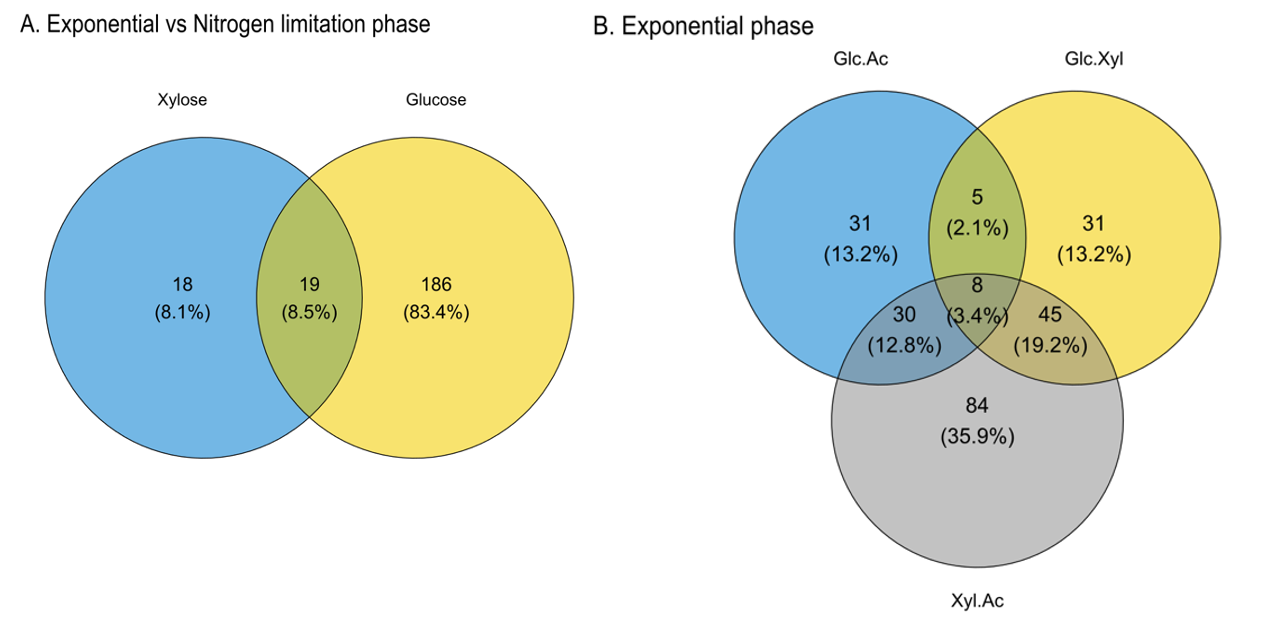

Supplement: S3 Fig — (A) Comparison between exponential growth (exp) and nitrogen limitation (Nlim) phase. (B) Comparison among substrates during exp phase. Comparison was made using μg/g of total protein. Pairs having adjusted p-value < 0.05 and log2 fold change > |1| were considered significantly differentially expressed. P value was adjusted for multiple comparisons (n = 3100) according to Benjamini & Hochberg (1995). (TIF) [file pcbi.1011009.s016.tif]

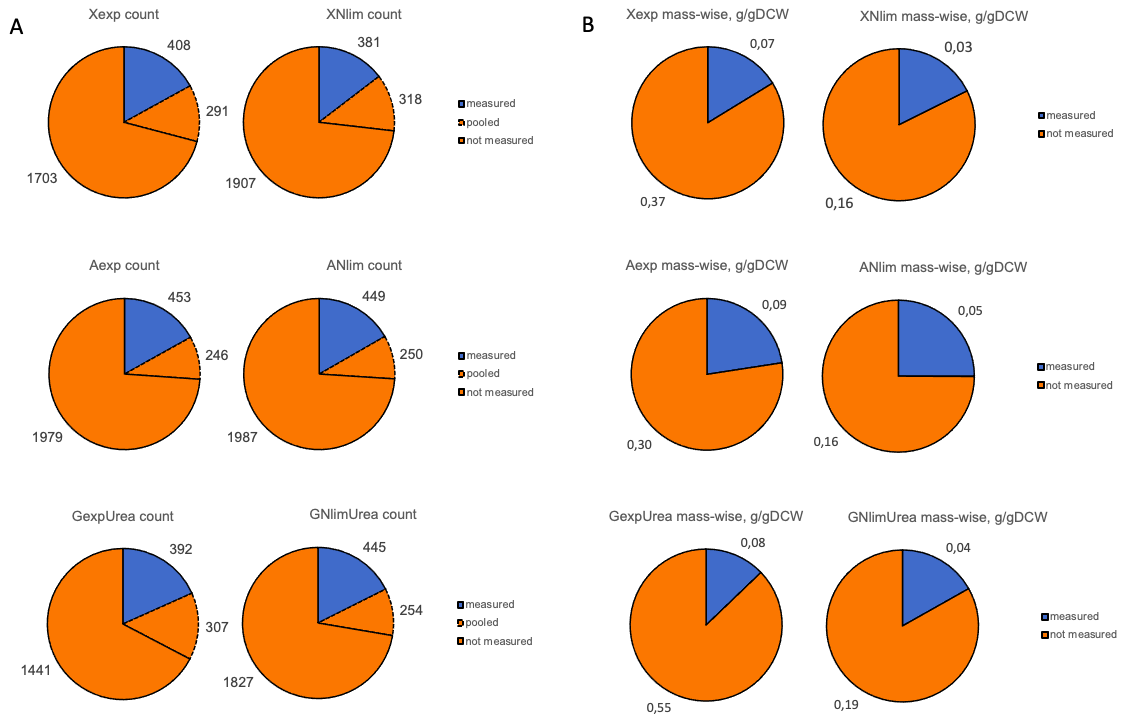

Supplement: S4 Fig — (A) Protein count as searched against the reference proteome database of R. toruloides strain NP11. (B) Mass-wise coverage of proteome in models (g_protein/g_DCW). (TIF) [file pcbi.1011009.s017.tif]

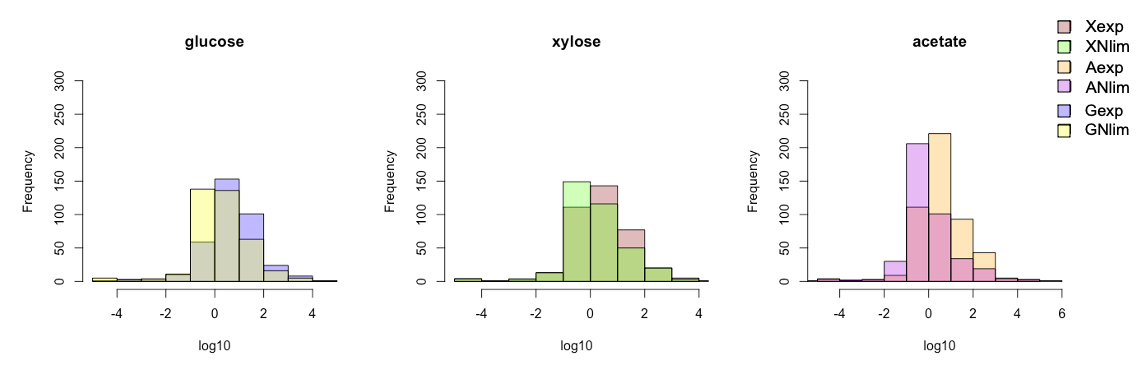

Supplement: S5 Fig — kapp calculated using fluxes from flux balance analysis on enzyme-constrained models of R. toruloides and measured enzyme absolute abundances. Frequency of kapp values represented in log10 scale. (TIF) [file pcbi.1011009.s018.tif]

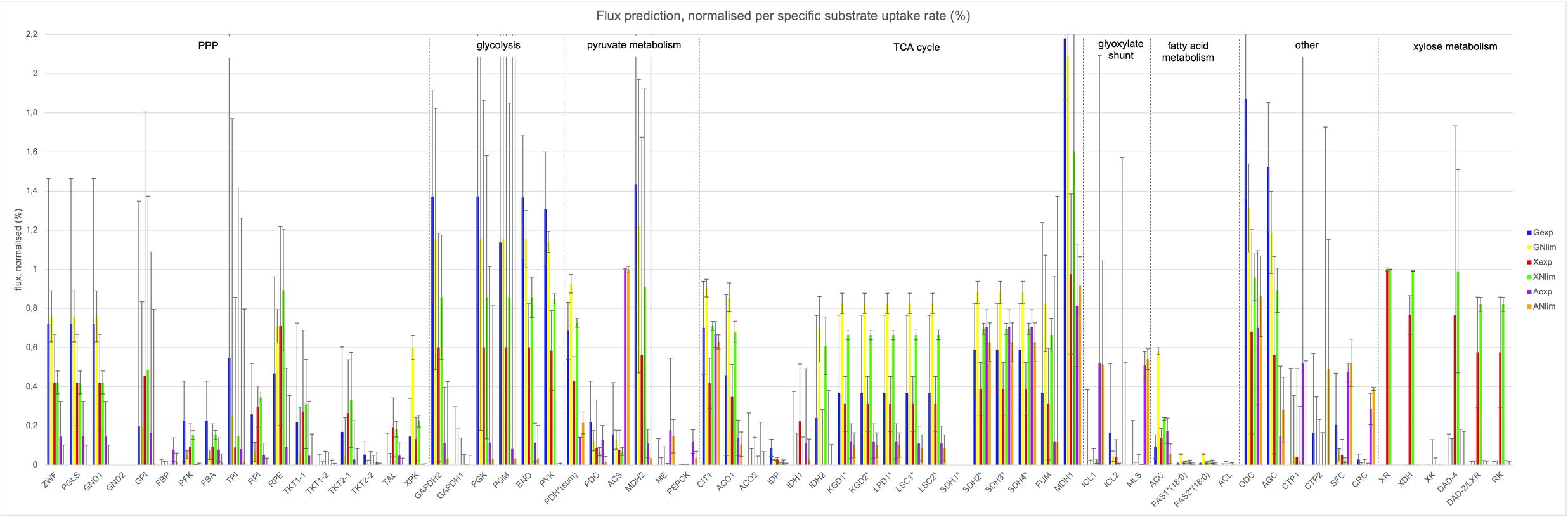

Supplement: S6 Fig — Fluxes are calculated using random sampling of the solution space with 2000 iterations (mmol/gDCW/h) on R. toruloides enzyme-constrained genome-scale models. Fluxes represent median values and are normalized by dividing flux with specific substrate uptake rate (representing % of carbon distribution). PPP: pentose phosphate pathway; TCA cycle: tricarboxylic acid cycle. Gene names and corresponding metabolic reaction IDs are included in S2 Table. (TIF) [file pcbi.1011009.s019.tif]

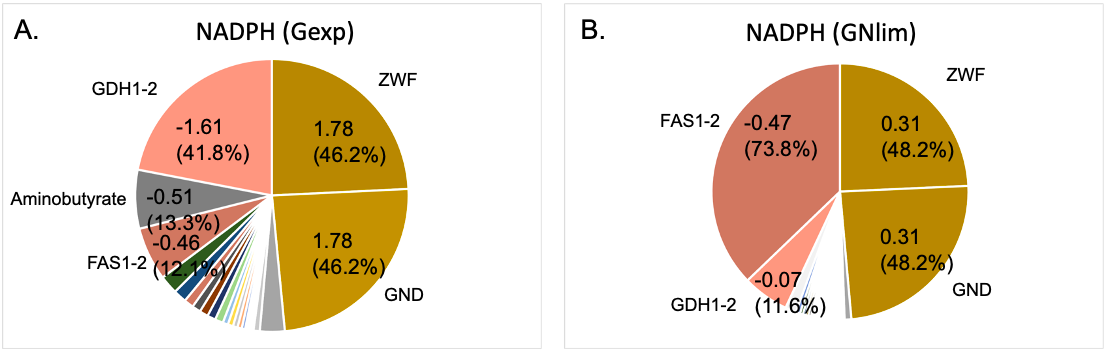

Supplement: S7 Fig — Fluxes are calculated using random sampling of the solution space with 2000 iterations (mmol/gDCW/h) on R. toruloides enzyme-constrained genome-scale models. Fluxes represent median values from flux sampling. Negative fluxes denote metabolite consumption, positive fluxes denote metabolite production. Percentage (%) denotes the flux divided by NADPH turnover (sum of absolute fluxes involving NADPH). Gene names and corresponding metabolic reaction IDs are included in S2 Table. (TIF) [file pcbi.1011009.s020.tif]

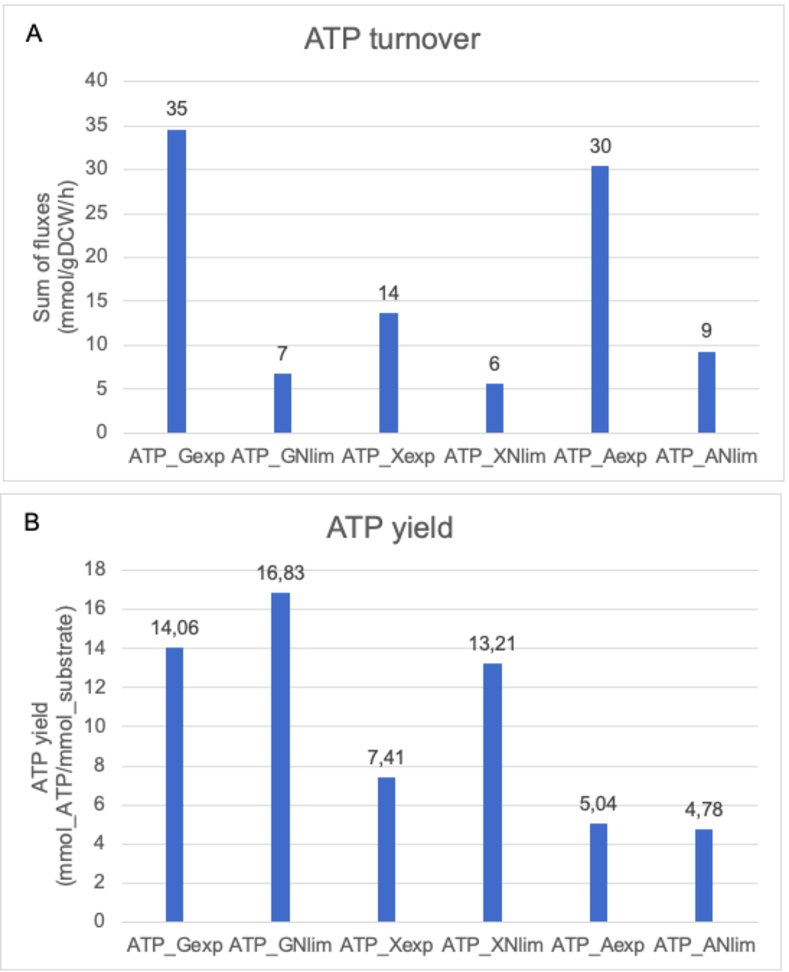

Supplement: S8 Fig — Predicted ATP turnover (mmol/gDCW/h) (A) and ATP yield (mmol_ATP/mmol_substrate) (B) in R. toruloides on three different carbon substrates–glucose (G), xylose (X) and acetate (A)–in a chemically defined medium at exponential growth (exp) and nitrogen limitation (Nlim) phases. ATP turnover is calculated as a sum of fluxes involving ATP. ATP yield is calculated as turnover divided by specific rate of substrate uptake. Fluxes are predicted using random sampling of the solution space with 2000 iterations (mmol/gDCW/h) on R. toruloides enzyme-constrained genome-scale models. Median flux values are used in calculations. (TIF) [file pcbi.1011009.s021.tif]

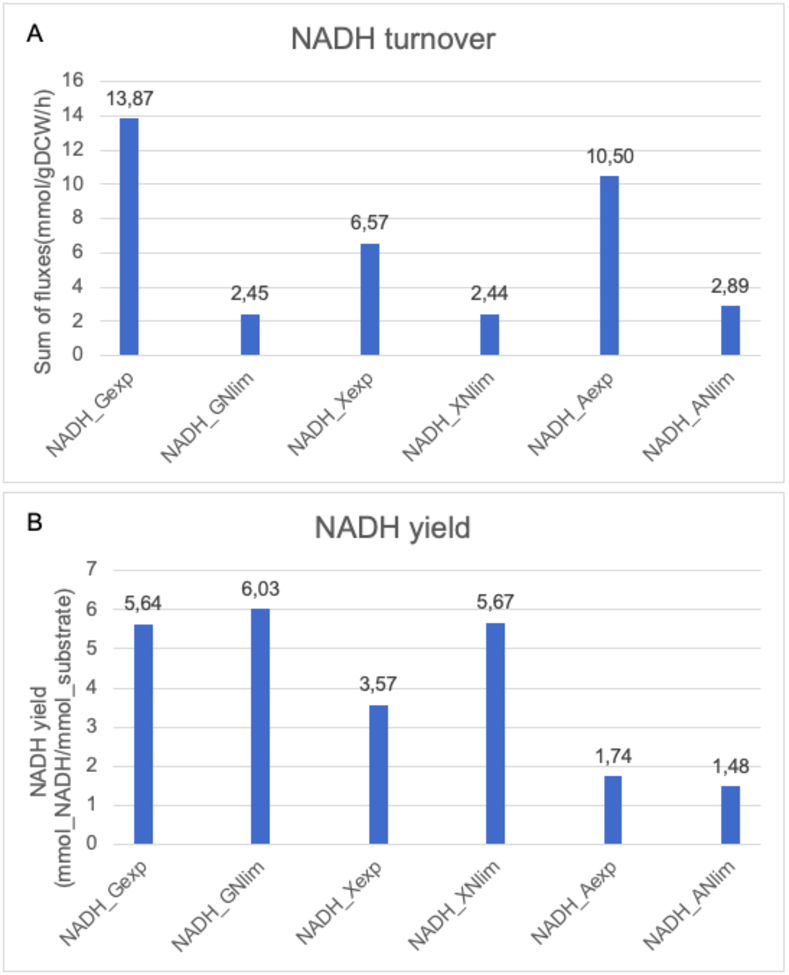

Supplement: S9 Fig — Predicted NADH turnover (mmol/gDCW/h) (A) and NADH yield (mmol_NADH/mmol_substrate) (B) in R. toruloides on three different carbon substrates—glucose (G), xylose (X) and acetate (A)–in a chemically defined medium at exponential growth (exp) and nitrogen limitation (Nlim) phases. NADH turnover is calculated as sum of absolute fluxes involving NADH. NADH yield is calculated as turnover divided by specific rate of substrate uptake. Fluxes are predicted using random sampling of the solution space with 2000 iterations (mmol/gDCW/h) on R. toruloides enzyme-constrained genome-scale models. Median flux values are used in calculations. (TIF) [file pcbi.1011009.s022.tif]

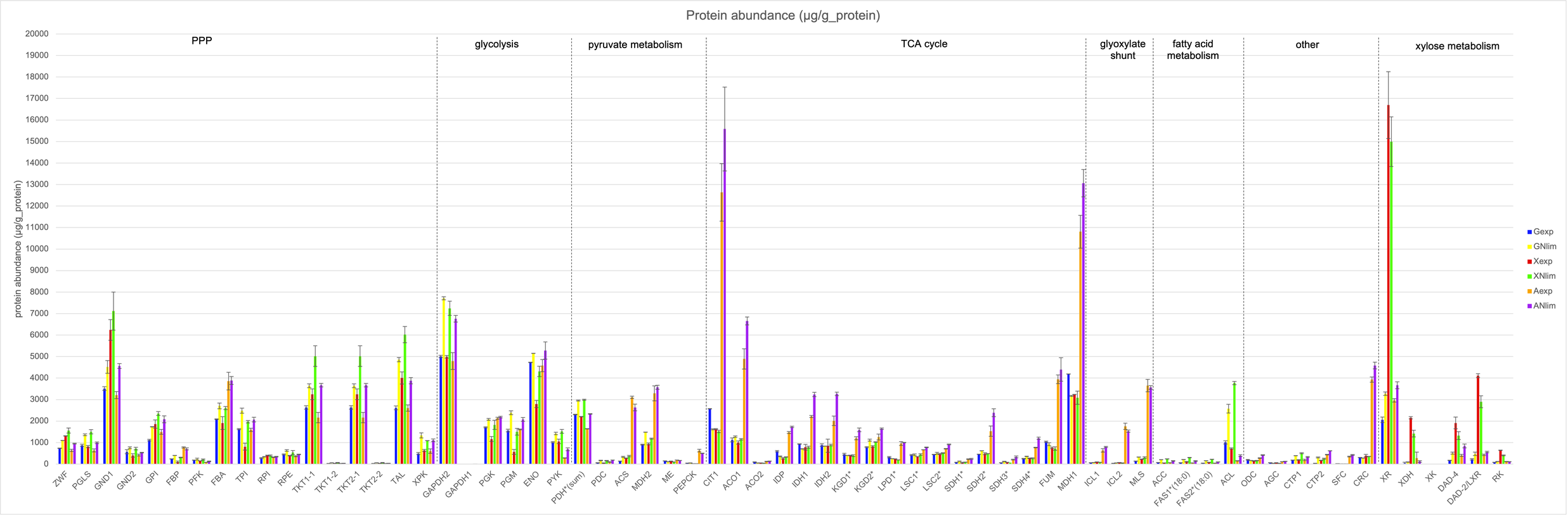

Supplement: S10 Fig — Absolute enzyme concentrations are calculated using total protein amount (TPA) quantification method. Results of duplicate experiments with SD are represented. PPP: pentose phosphate pathway; TCA cycle: tricarboxylic acid cycle. Full names of gene abbreviations are included in S2 Table. (TIF) [file pcbi.1011009.s023.tif]

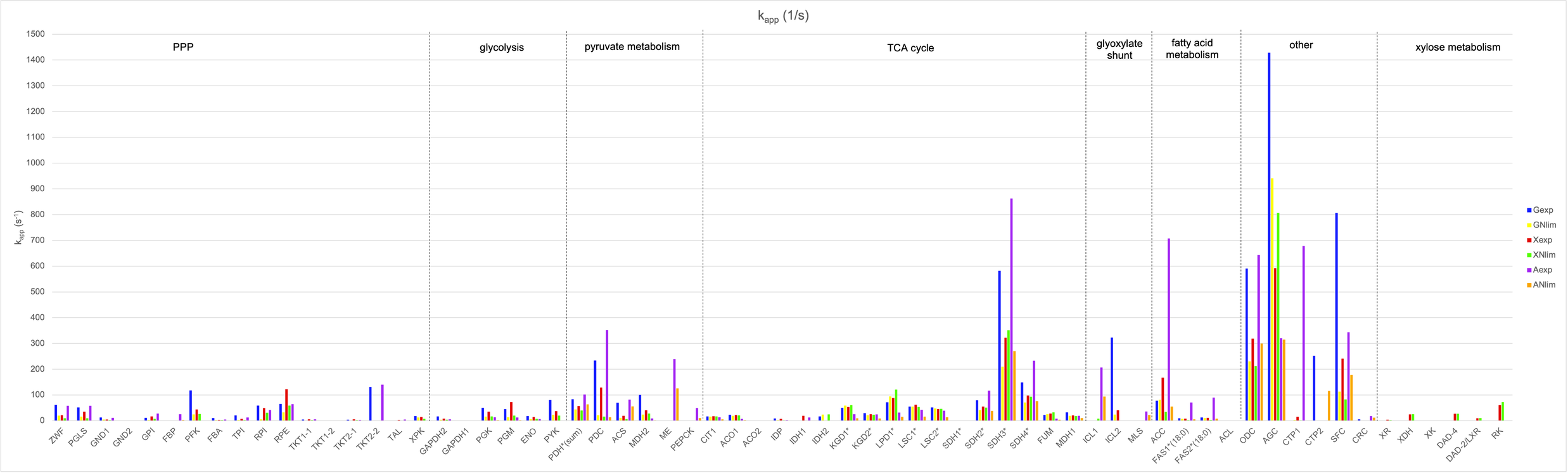

Supplement: S11 Fig — kapp calculated using fluxes from flux balance analysis on enzyme-constrained genome-scale models of R. toruloides and measured enzyme absolute abundances. PPP: pentose phosphate pathway; TCA cycle: tricarboxylic acid cycle. Full names of gene abbreviations are included in S2 Table. (TIF) [file pcbi.1011009.s024.tif]

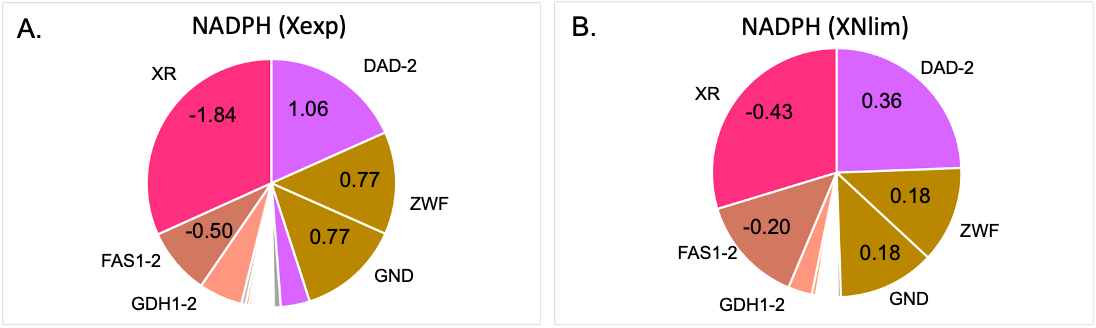

Supplement: S12 Fig — Fluxes are calculated using random sampling of the solution space with 2000 iterations (mmol/gDCW/h) on R. toruloides enzyme-constrained genome-scale models. Fluxes represent median values from flux sampling. DAD-2/LXR is considered NADP-dependent. Negative fluxes denote metabolite consumption, positive fluxes denote metabolite production. Gene names and corresponding metabolic reaction IDs are included in S2 Table. (TIF) [file pcbi.1011009.s025.tif]

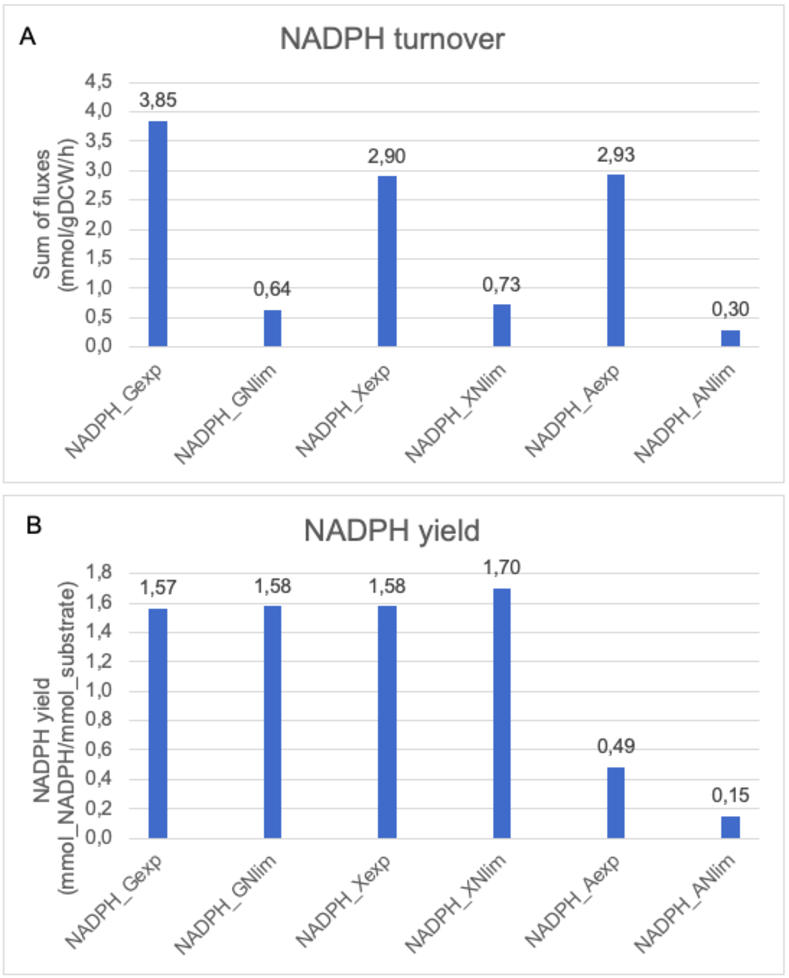

Supplement: S13 Fig — Predicted NADPH turnover (mmol/gDCW/h) (A) and NADPH yield (mmol_NADPH/mmol_substrate) (B) in R. toruloides on three different carbon substrates—glucose (G), xylose (X) and acetate (A)–in a chemically defined medium at exponential growth (exp) and nitrogen limitation (Nlim) phases. NADPH turnover is calculated as sum of absolute fluxes involving NADPH. NADPH yield is calculated as turnover divided by specific rate of substrate uptake. Fluxes are predicted using random sampling of the solution space with 2000 iterations (mmol/gDCW/h) on R. toruloides enzyme-constrained genome-scale models. Median flux values are used in calculations. (TIF) [file pcbi.1011009.s026.tif]

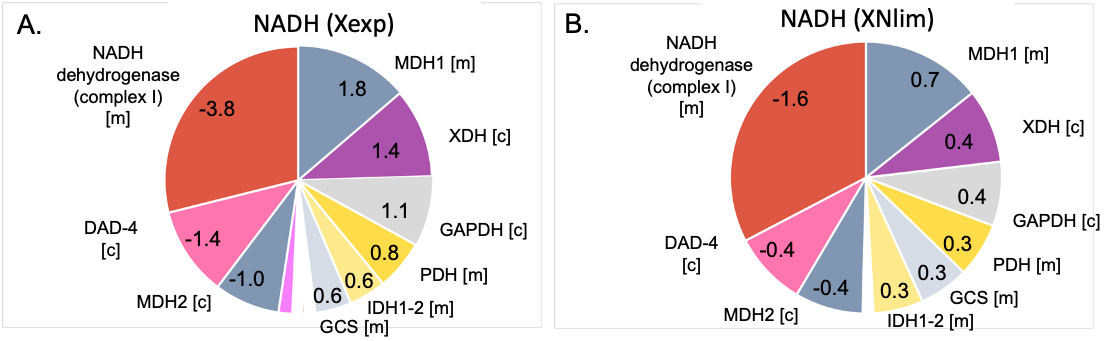

Supplement: S14 Fig — Fluxes are calculated using random sampling of the solution space with 2000 iterations (mmol/gDCW/h) on R. toruloides enzyme-constrained genome-scale models. Fluxes represent median values from flux sampling. DAD-2/LXR is considered NADP-dependent. Negative flux denotes metabolite consumption, positive flux denotes metabolite production. Letters [m] and [c] denote compartments of cytoplasm and mitochondria. Gene names and corresponding metabolic reaction IDs are included in S2 Table. (TIF) [file pcbi.1011009.s027.tif]

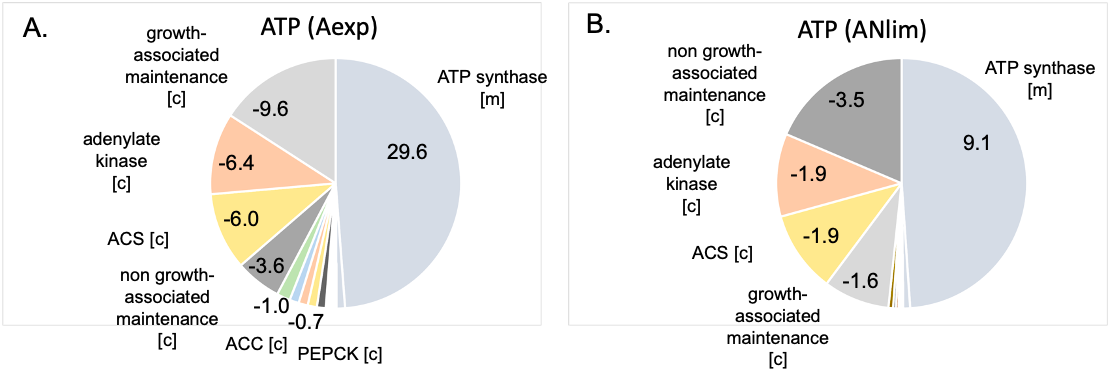

Supplement: S15 Fig — Fluxes carrying ATP in R. toruloides on acetate-(A) based chemically-defined medium at exponential growth (exp) (A) and nitrogen limitation (Nlim) (B) phases (mmol/gDCW/h). Fluxes are calculated using random sampling of the solution space with 2000 iterations (mmol/gDCW/h) on R. toruloides enzyme-constrained genome-scale models. Fluxes represent median values from flux sampling. Negative flux denotes metabolite consumption, positive flux denotes metabolite production. Letters [m] and [c] denote compartments of cytoplasm and mitochondria. Gene names and corresponding metabolic reaction IDs are included in S2 Table. (TIF) [file pcbi.1011009.s028.tif]

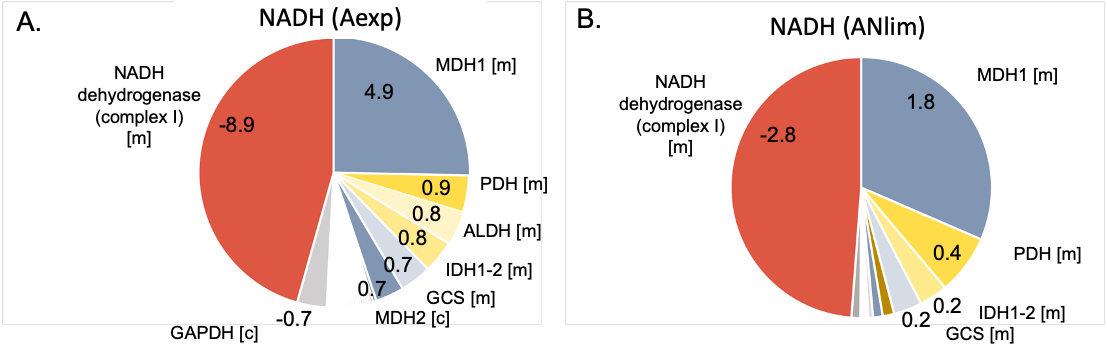

Supplement: S16 Fig — Fluxes are calculated using random sampling of the solution space with 2000 iterations (mmol/gDCW/h) on R. toruloides enzyme-constrained genome-scale models. Fluxes represent median values from flux sampling. Negative flux denotes metabolite consumption, positive flux denotes metabolite production. Letters [m] and [c] denote compartments of cytoplasm and mitochondria. Gene names and corresponding metabolic reaction IDs are included in S2 Table. (TIF) [file pcbi.1011009.s029.tif]

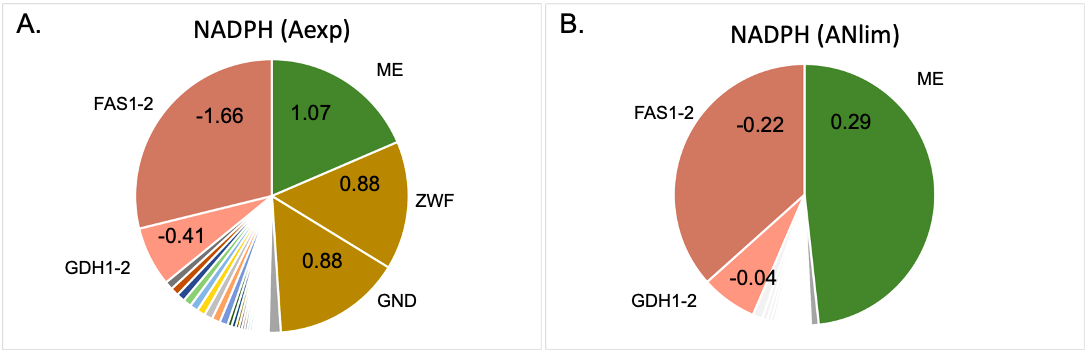

Supplement: S17 Fig — Fluxes are calculated using random sampling of the solution space with 2000 iterations (mmol/gDCW/h) on R. toruloides enzyme-constrained genome-scale models. Fluxes represent median values from flux sampling. Negative flux denotes metabolite consumption, positive flux denotes metabolite production. Gene names and corresponding metabolic reaction IDs are included in S2 Table. (TIF) [file pcbi.1011009.s030.tif]

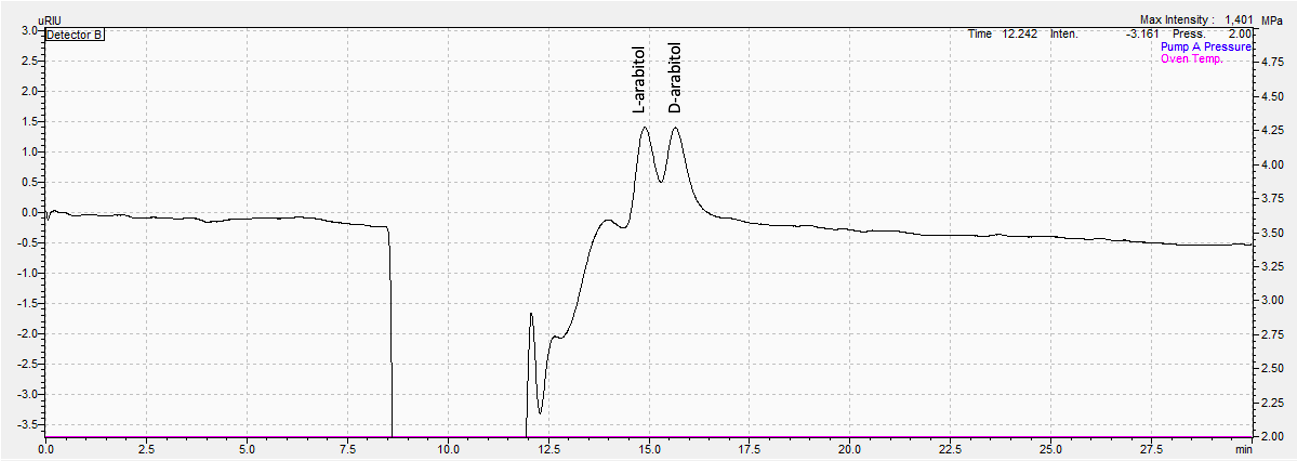

Supplement: S18 Fig — Figure represents retention times for arabinitol separation in Chiralpak column, at 20°C, hexane-ethanol (70:30, v/v) mixture. (TIF) [file pcbi.1011009.s031.tif]
