# Supplementary material for: Genome-scale metabolic modeling reveals metabolic trade-offs associated with lipid production in Rhodotorula toruloides
Source: PLoS Comput Biol. 2023 Apr 26;19(4):e1011009. doi: 10.1371/journal.pcbi.1011009 (PMC10204961; doi:10.1371/journal.pcbi.1011009)
Supplement: Attachment — Submitted filename: Responses.docx [file pcbi.1011009.s032.docx]

We would like to thank both reviewers for their contribution. We find the issues and comments raised by reviewers constructive, and we prepared a revised manuscript taking these suggestions into account. Below are point-to-point remarks regarding the issues brought up by the reviewers. Please, note that reviewers' questions are marked in blue, and line numbers are referring to a marked-up copy of the revised manuscript.

# Reviewer #1:

# Major concerns: 1. Page 4, line 73-75, the authors need to also mention the ATP cost to convert acetyl-CoA to malonyl-CoA. This also appears again on page 16, line 359.

Line 77 in the main text: Added ATP cost for conversion of acetyl-CoA to malonyl-CoA.

Also, added later in the section “Growth on glucose”, line 448 in the main text.

2. Page 5, line 93-98, can the authors provide a comment (or perhaps a more explicitly statement) on if xylulokinase is not encoded in the genome, or if it is encoded but not expressed?

Xylulokinase is encoded in the genome. This has been inserted to line 98 in the main text.

3. Page 7, line 134-136, this sentence contradicts a later statement which is that separate versions of the model were built for each growth condition.

Lines 141-143 in the main text: modified to state that separate versions of the model were built for each growth condition.

4. Page 7, line 154, it would be helpful to list all “parameters” here as an overview for what to be discussed.

Lines 161-162 in the main text: Instead of writing “parameters”, inserted “production profile, specific growth rate, lipidomics and total protein measurements” that were discussed further in that section.

5. Description of “mineral medium” is unclear. What are in “1 mL/L vitamin solution”? What are the differences compared to the commonly used Yeast Nitrogen Base (YNB) media for yeasts?

Changed “mineral medium” to “chemically defined medium” throughout the text, because it is a general term for chemically defined media that was used in the study.

Description of “chemically defined medium” clarified by listing all components instead of “1 mL/L vitamin/trace metal solution”. The reason for writing “1 mL of solution” was because we used a concentrated stock solution to prepare the medium, but now the confusion is eliminated by listing only the final concentrations of compounds.

Also, we have referenced the original publication. We did not use YNB, but another popular CDM that is often referred to as Verduyn medium ([**https://doi.org/10.1002/yea.320080703**](https://doi.org/10.1002/yea.320080703)), developed to support yeast biomass concentrations up to 10 g/L in aerobic, glucose-limited cultures. In comparison to YNB, Verduyn medium contains more inositol, but does not contain riboflavin (B2) and folic acid (B9) (in detail reviewed here: [**https://doi.org/10.1002/yea.3461**](https://doi.org/10.1002/yea.3461)). There are 1080 citations in Scopus for the original publication of Verduyn medium, including recent (Elsemman et al. 2022, <https://doi.org/10.1038/s41467-022-28467-6>) and older ones (Vemuri et al. 2007, https://doi.org/10.1073/pnas.0607469104).

Lines 896-907 in the main text: modified “Seed cultures were grown on chemically defined medium according to Verduyn (3.0 g/L KH2PO4, 0.5 g/L MgSO4·7H2O, 15 mg/L EDTA, 4.5 mg/L ZnSO_4_⋅7H_2_O, 0.3 mg/L CoCl_2_⋅6H_2_O, 1 mg/L MnCl_2_⋅4H_2_O, 0.3 mg/L CuSO_4_⋅5H_2_O, 4.5 mg/L CaCl_2_⋅2H_2_O, 3 mg/L FeSO_4_⋅7H_2_O, 0.4 mg/L Na_2_MoO_4_⋅2H_2_O, 1 mg/L H_3_BO_3_, 0.1 mg/L KI, 0.05 mg/L biotin, 1 mg/L calcium pantothenate, 1 mg/L nicotinic acid, 25 mg/L inositol, 1 mg/L thiamine HCl, 1 mg/L pyridoxine HCl, 0.2 mg/L *para*-aminobenzoic acid [51]) supplemented with a sole carbon source of 18.2 g/L glucose, 20 g/L xylose or 20.0 g/L acetic acid and 5 g/L (NH4)2SO4 in duplicate shake flasks at 200 rpm and 30°C for 24 h.”

6. Page 9, line 196-201, the two sentences contrast each other. First sentence mentions similarities whereas the second sentence mentions differences between strains. The authors need to re-write and add more explanations to these sentences to prevent confusion. “The final fatty acid compositions were in agreement with the previous data reported on glucose and xylose [8]. In our study, the growth phase-dependent differences in compositions were linked to PUFAs, mainly linoleate (C18:2), while in the previous study [8] it was linked mostly to palmitate (C16:0), which possibly reflect the fact that different R. toruloides strains were used in these studies.”

During nitrogen limited growth phase (Nlim), palmitate and oleate increased in [8] as well as in our study, representing the largest part of lipidome. With the sentence we intend to convey that in our study linoleate increased more, while in [8] palmitate increased more at Nlim. We have rephrased the sentence for clarity.

Lines 240-246 in the main text: modified to “The distribution of different types of fatty acids was in agreement with the previous data reported on glucose and xylose [8], whereby oleate and palmitate where the most abundant at the end of batch cultivation. Notwithstanding the general agreement between both studies, in our study the PUFAs, mainly linoleate (C18:2) increased more during Nlim, while in the previous study [8] palmitate (C16:0) increased more during lipid accumulation.”

7. Page 9, line 203 (plus any other instances of the same type), in the unit of biomass yield “gDCW/g_carbon”, what does “g_carbon” refer to? Is this “g_substrate” (e.g., carbon substrate of glucose)? Using “g_substrate” is better since “g_carbon” might mislead the readers for only the carbon content of substrate.

Unit of biomass yield refers to carbon substrate of glucose, xylose or acetate. For clarity, we have now changed yield units to “g_substrate” instead of “g_carbon” throughout the text to not mislead for only the carbon content of substrate.

Also, “specific carbon uptake (rate)” has been changed to “specific substrate uptake (rate)” and “r_substrate” instead of “r_carbon” in manuscript text and all supplementary files.

Finally, units of yields of cofactors (ATP, NAD, NADPH) have been modified to “mmol_ATP/mmol_substrate” instead of “mmol_ATP/mmol_carbon”. Legends of supplementary figures have also been modified in supplementary files and included in the end of manuscript file.

8. Page 10, the author mentioned that “However, we were able to measure only 68.5% of carbon during the Nlim phase (S1 Table). Likely, it was due to the fact that R. toruloides strain CCT 7815 was making cell aggregates in the minimal mineral glucose-based medium.”. This is a very important observation and lesson for future researchers in working with R. toruloides. Could the authors further elaborate how “making cell aggregates” lead to incomplete mass balance in measurements? This might be unclear to readers, especially those without extensive experience working with the organism experimentally.

Before sampling, optical density of cultures was determined via spectrophotometer. Biomass optical density data were calibrated by gravimetric cell mass measurements (lines 945-946 in the main text).

We have now referenced Haaber et al. 2016 and Koch 1970 to explain that optical density measurement is based on light scattering by the cells in the liquid and this is correlated to cell numbers under equal size and distribution assumption. If this assumption is not met due to aggregation, optical density measurements will underestimate the actual number of cells in the liquid.

Lines 277-282 in the main text: inserted “Mass balance calculation took into account glucose uptake, carbon dioxide production and biomass (in C-mol). As our biomass measurements were based on optical density, which relies on the assumption that cells are evenly distributed and of equal size [24,25], it may underestimate the actual cell concentration in liquid culture when aggregates are formed.”

The authors then moved on to mention that: “The growth curves were highly similar when using ammonium sulfate or urea (S1A Figure), and therefore, further analysis with glucose was carried out with cells using urea as a nitrogen source.” The authors need to make it clearer for what results correspond to what nitrogen source (i.e., ammonium and urea). This includes: (i) exp or N-lim phase, (ii) glucose only or not (which is already clear), (iii) applied for which data (i.e., on Fig. 1 and for proteomics and/or flux data), and (iv) recorded in Supplementary Materials.

Lines 287-290 in the main text: modified to “Therefore, results exclusively in the glucose condition (both exp and Nlim growth phases), including the proteomics and metabolic flux data presented in main text and Supplementary Materials, belong to experiments in which we used urea as a nitrogen source.”

9. Line 241-245, PCA has also been done for R. toruloides using transcriptomics data (Jagtap et al., 2021, <https://doi.org/10.1007/s00253-021-11549-8>). It would be informative if the authors can provide few sentences comparing results of PCA using proteomics vs. transcriptomics. Do similar patterns arise in both? Are there any noticeable differences?

We have provided additional sentences comparing our proteomics results with transcriptomics results; found similar patterns between both studies.

Lines 312-320 in the main text: inserted “PC analysis has previously been done for *R. toruloides* strain IFO 0880 comparing gene expression during the exponential growth phase on rich medium containing sole carbon substrate, similarly as in our study [3]. PC1 using transcriptomics revealed distinct expression patterns on acetate-grown as compared to glucose- and xylose-grown cells, agreeing with the proteomics results obtained in our study. The only noticeable difference was that PC2 in [3] separated exp phase from glucose to xylose. In our study, PC2, describing 34% of the variation in our data, separated mainly exp and Nlim conditions in the same way on glucose and xylose (**Figure 2A**).”

10. In page 15, where and how are the kcat values obtained for R. toruloides, given that this is a non-model organism whose reaction kinetic parameter data is not readily available? This information is important to be (at least briefly) mentioned in the main text so that the readers have a perspective on what (organism/data) sources are enzymatic efficiency parameters taken from.

This is a very good point. We have brought out how the k_cat_ values were obtained before discussing modelling results. We added a few sentences explaining where and how the k_cat_ values for *R. toruloides* were obtained. This also refers to comment No. 22 from the Reviewer.

In that regard, the Discussion section has been clarified by removing sentences that explain k_cat_ origin. It is unchanged regarding the sentence “not fully matched k_cat_ values can notably increase the prediction errors in enzyme-constrained genome-scale models [45]” in order to bring a further perspective to the readers as to what might be the possible consequences of using this approach to selecting the k_cat_ values. For future work, we are planning to predict k_cat_ values based on protein structure, but this is out of scope for the current manuscript.

Lines 400-404 in the main text: inserted “... (S4 Table), which enabled GECKO Toolbox to assign their k_cat_ values. At first, BRENDA was queried for exact matching reaction, substrate and organism. But as kinetic parameter data for non-model organisms such as *R. toruloides* are not readily available, GECKO Toolbox step-wise relaxes the stringency when matching EC number, organism and substrate, to assign reasonable estimates of k_cat_ values [30].”

11. In page 15, the following sentence does not make sense: “Convex Basis sampling algorithm [28] with 2000 sampling points and a random set of objective functions to be maximized to evaluate flux variability”. Why would sampling of a solution space require an optimization objective?

The sentence has been simplified and the phrase “random set of three reactions was given random weights and the sum of these were parsimoniously maximized” is moved to the Methods section (lines 1164-1165 in the main text).

For clarification, the objective function is not a single objective for the whole sampling procedure. For each sample, a random set of three reactions is chosen and maximized with random weights, aiming to describe the surface of the solution space (RAVEN Toolbox, Agren et al. 2013, PLOS Comp. Biol.).

Line 410-412 in the main text: modified “Next we used Flux Balance Analysis [29] to simulate intracellular flux patterns and random of the solution space [30] with 2000 sampling iterations to evaluate flux variability (**S4 Dataset**).”

Lines 1166-1174 in the main text: modified “Flux variability was estimated with random sampling of the solution space with 2000 sampling iterations for each condition (ie., ec-model). For each sample, a random set of three reactions was given random weights and the sum of these were parsimoniously maximized to explore the constraint solution space [32], considering 1% variability from maximal growth rate and substrate uptake rate, 10% variability from predicted carbon dioxide production and oxygen consumption rate, 10% variability from measured by-product rates, 10% variability from protein pool, and 1% variability from NGAM, as specified in *analyze_ecRhtoGEM*.”

12. Page 16, line 341, “apparent” (or “effective”) should be used instead of “actual” to describe kapp to be consistent with previous publications. Line 345, do the authors mean “in vivo” instead of “in silico” when describing the kapp?

Line 419 in the main text: as suggested, the term “apparent” is now used instead of “actual”

Line 426 in the main text: We mean “*in silico* k_app_ values” - model-predicted fluxes and measured protein concentration, whereas *in vivo* would refer to experimentally measured values.

13. Page 16, line 343-344, the authors set model parameters as follow: “kapp is set by the in vitro enzyme turnover number kcat”. This might omit kapp > kcat cases being valid because of the in vivo enzyme activity enhancement effect. There are examples on in vivo kapp (calculate using similar methods to that used in this manuscript) available in the literature for the authors to read.

It is important to note that the k_app_ that we calculate are based on the fluxes that are predicted by the ec-models.

Fluxes in the ec-models are constraint by a combination of k_cat_ values and measured protein concentrations (for those reactions that we have such information of course).

- therefore, if model-flux ≤ kcat * [E], then [E] / flux = kapp ≤ kcat.

- kapp > kcat would mean that the model-predicted flux would have violated its constraint.

Cases where we found kapp = kcat, it is indeed possible that *in vivo* this might have been kapp > kcat, but this we are not able this capture in our modelling framework. For clarity, we have removed statement “upper limit of calculated k_app_ is set by the in vitro enzyme turnover number k_cat”_.

Lines 417-418 in the main text: modified “From the simulated flux values, we calculated apparent enzyme catalytic activities (k_app_) as ratio of model-predicted fluxes and measured protein concentration.”

Lines 421-424 in the main text: inserted “As we used model-predicted fluxes that were constrained by k_cat_ values in the ec-model, the k_app_ values that we obtained cannot be higher than the k_cat_ value, which means that we cannot capture any potential *in vivo* enzyme activity enhancement effect. Regardless, ...”

Line 422 in the main text: deleted “Consequently, the upper limit of the calculated kapp is set by the in vitro enzyme turnover number kcat.”

14. In calculating kapp, the authors should consider using parsimonious FBA solution to represent minimally required flux activity instead of using flux sampling. This is because sampling will generate noise which mean that the kapp calculated from sampled flux is unreliable for low flux pathways, such as for fatty acid oxidation as noted by the authors: “Some of the lowest kapp values in acetate condition were associated with fatty acid degradation and beta oxidation metabolic pathways.”. Fatty acid degradation function under these conditions is not clear and thus their fluxes calculated by flux sampling are unreliable anyway.

We appreciate the importance of using parsimonious solutions, therefore we have included this as part of the random sampling procedure. For each sample, after obtaining the optimal value for the random objective function, the number of fluxes is minimized (RAVEN Toolbox uses a minFlux flag in its solveLP function).

In our opinion, parsimonious random sampling is preferred over mere parsimonious FBA, as pFBA would yield only one potential solution with minimal fluxes, while it is near-inevitable that alternative optimal solution exist considering the size of the network. Parsimonious random sampling provides for flux distributions.

Firstly, RAVEN Toolbox (Angren et al. 2013, PLOS Comp. Biol.) offers a minFlux parameter to minimize the absolute sum of fluxes and thus get rid of loops during the flux balance analysis. The principle of minFlux and pFBA is the same. We used minFlux in every FBA during the flux sampling*.

Secondly, pFBA gives only one potential solution with minimal fluxes, but considering the size of the network it is feasible that alternative optimal solutions exist, particularly allowing some tolerance in finding the most minimal required flux activity. Therefore, flux sampling, with nested minimization of fluxes, provides for flux distributions that could have been underlying the observed phenotype, while taking into consideration both: (i) that they are unlikely to contain numerous futile cycles and non-optimal pathways, and (ii) at the same time acknowledging that more enzymes might be active than the absolute minimum required (for instance, due to thermodynamics and regulation).

As such, our simulation results are not a mere consequence of noise in the random sampling, and we feel therefore confident to retain the statements about fatty acid degradation.

*Implementation of minFlux at *analyze_ecRhtoGEM*:

rsmatrix_Xexp=rs(ecModelP_XexpTmp,2000,true,true,true,goodRxns,true);

where the 7th parameter corresponds to minFlux (usage: solutions=randomSampling(model,nSamples,replaceBoundsWithInf,supressErrors,showProgress,goodRxns,minFlux))

15. In page 17, line 372, the authors need to provide evidence from their own data or from the literature on phosphoketolase pathway activity. At least, the authors should comment and follow up on: (i) enzyme expression (for all of the steps), (ii) FBA simulation without phosphoketolase being active to confirm no alternative pathways exist to functionally replace it, and (iii) definite resolution of phosphoketolase pathway would require a follow-up metabolic flux analysis study (using isotopically labeled tracer). This is necessary so as not to mislead readers on being confident about phosphoketolase pathway activity.

In page 18, the authors comment on the ATP citrate lyase pathway to be a functional equivalent to phosphoketolase in producing cytosolic acetyl-CoA. Why is this pathway not being active instead of phosphoketolase? This might be resolved if the authors perform simulation with phosphoketolase or ATP citrate lyase being inactive and analyze the results.

New results have been added in lines 480-486 in the main text. Regarding evidence on phosphoketolase pathway activity, we have added data on protein expression in which exp and Nlim phases were compared and supported XPK activity during lipid accumulation.

We have clarified about which metabolic steps are part of the phosphoketolase pathway to inform readers that we have evaluated the possibility of acetate kinase (ACK) being present in the pathway, as reported in *R. toruloides* models for strain IFO 0880 (Dinh et al. 2019, Kim et al. 2021), while phosphotransacetylase (PTA) reaction was included for strain NP11 in rhtoGEM (Tiukova et al. 2019). We have compared fluxes in both scenarios, and they were highly similar. The biggest difference that was brought in by ACK was reflected in the ATP balance. The net ATP difference in both results turned out to be negligible, therefore we concluded to continue with the flux analysis based on PTA presence. Models with ACK are available under *acetateKinase* branch on ecRhtoGEM Github repository. Flux results are available from S5 Dataset.

Lines 480-486 in the main text: inserted “consistent with a significant upregulation of phosphoketolase (XPK) on proteome level (apval. 0.043, **S2 Dataset**). While it is not known, which route of XPK enzyme in combination with a phosphotransacetylase (PTA) or an acetate kinase (ACK) is used in *R. toruloides* strain CCT 7815, we compared the fluxes of both possible scenarios (**S4 Dataset**, **S5 Dataset**). As the results were highly similar, further flux analysis was carried out based on a metabolic route where PTA is active.”

We would also like to clarify that we did not want to mislead readers that the XPK pathway was the only route for cytosolic acetyl-CoA production. We wanted to show which of the pathways are preferred, and in our study it turned out to be XPK. When we performed simulations with XPK blocked, it showed that pyruvate decarboxylase and ACL routes can replace XPK in producing cytosolic acetyl-CoA during Nlim and produce a feasible result (S6 Dataset).

To explain this finding, we can argue that XPK saves one CO_2_ molecule and at least one equivalent of ATP per pyruvate as compared to pyruvate decarboxylase or ATP citrate lyase pathways (addressed in the Discussion section). However, the intrinsic mechanisms we do not know.

Lines 488-491 in the main text: inserted “The pyruvate decarboxylase and ACL, which exist as alternative pathways for producing cytosolic acetyl-CoA during lipid accumulation, were activated only when we blocked the XPK pathway (**S6 Dataset**).”

Lines 771-776 in the main text: inserted “Predicted acetate kinase activity demonstrated certain robustness towards the preference for the XPK pathway (**S5 Dataset**). XPK pathway is considered an attractive option for generating cytosolic acetyl-CoA because it is more energy and carbon efficient. It circumvents one molecule of CO_2_ lost per pyruvate and two equivalents of ATP consumed compared to the *PDH bypass* route (PDC-ALDH and ACS).” I

And lastly, we are planning a larger follow-up study to investigate the activity of the phosphoketolase pathway and other metabolic routes, but that, irrespectively of whether it includes MFA or other method, would be outside the scope of the current manuscript.

16. Page 21, line 443-445, it is not clear why this is mentioned: “However, protein levels of L-xylulose reductase (EC 1.1.1.10, RHTO_00373) and L-arabinitol dehydrogenase (EC 1.1.1.12, RHTO_01629) were also 10-fold upregulated during growth on xylose versus other substrates.”

Sentence in line 547 was mentioned to support the choice of one of them for the alternative new pathway.

To clarify text, the sentence about protein levels is now moved after the sentence about LXR function from the literature. Also, part about LAD enzyme (Lines 548-556) is removed because it was, indeed, irrelevant.

Line 547 in the main text: deleted “However, protein levels of L-xylulose reductase (EC 1.1.1.10, RHTO_00373) and L-arabinitol dehydrogenase (EC 1.1.1.12, RHTO_01629) were also 10-fold upregulated during growth on xylose versus other substrates”

Lines 548-556 in the main text: modified and moved “In support of this mechanism, protein levels of L-xylulose reductase (EC 1.1.1.10, RHTO_00373) were 10-fold upregulated during growth on xylose versus other substrates.”

17. Page 22, line 459-461, the authors mentioned: “In support of this mechanism, the metabolic model could only predict a feasible solution of LXR/DAD-2 only using NADP(+) as the cofactor.” Was this simulation performed with enzyme constraint (with kcat or kapp < kcat being active)? The authors need to also perform simulation with FBA (i.e., without fluxes constrained by enzyme levels) to assess if infeasibility is due to metabolic demand or due to enzyme level constraining flux. The authors might want to re-calculate and use a new kapp value if simulation with FBA is indeed feasible.

The inability to grow on NADH was a problem with our analysis pipeline, which we have now resolved (revision.m). Now, flux distributions with NADH or NADPH are highly similar, except for NADPH regeneration (S4 Dataset, S7 Dataset).

Lines 568-571 in the main text: inserted “While it is not known, which cofactor of DAD-2/LXR enzyme is operational in *R. toruloides* strain CCT 7815, we analyzed the fluxes of both possible scenarios. Both simulation results were highly similar, with a difference in where NADPH was regenerated.”

Line 571 in the main text: deleted “In our study, the LXR/DAD-2 was specific for NADP(+)/NADPH. In support of this mechanism, the metabolic model could only predict a feasible solution of LXR/DAD-2 only using NADP(+)/NADPH as the cofactor.”

Lines 573-575 in the main text: modified “In a scenario when DAD-2/LXR was NADP-dependent, during both exp and Nlim growth phases between 46-49% of carbon derived from xylose was directed via glucose 6-phosphate isomerase (GPI) in a reverse direction to the glycolytic flux.”

Lines 578-579 in the main text: inserted “Alternatively, up to 88% of xylose-derived carbon was directed via oxPPP (S7 Dataset).”

The new pathway was preferred, but the flux of oxPPP ‘’could be” compensated, keeping in mind that there were two feasible solutions in regard to the use of cofactor for DAD-2/LXR.

Lines 834-836 in the main text: modified “In comparison to our previous work [1], the flux of PPP could be compensated by the amount of carbon channeled via the alternative xylose pathway.”

18. Page 24, line 512-513, isn’t the decrease in ACC flux simply due to the fact that lipid still need to be produced even in exp phase? This means that the lipid produced for the much higher growth rate in exp phase is higher in absolute amount compared to that in N-lim phase.

This is a good suggestion, which also corresponds to our data.

Lines 631-633 in the main text: inserted “Using the rate of lipid production, which decreased during Nlim phase, it can be explained that the lipid production in absolute amounts was higher during the exp phase to sustain the growth together with moderate lipid production (**S1 Table**).”

19. Page 24, line 526, is this ME the cytosolic one? This needs to be clarified since there is also mitochondrial ME.

It was cytosolic ME that provided all NADPH during Nlim in acetate condition.

Line 649 in the main text: inserted “cytosolic ME”

20. Page 26, line 563, what are the other “alternative enzymes of the nitrogen assimilation pathway”? This needs to be clarified since GDH is commonly known to be the only nitrogen fixation pathways with ammonium as nitrogen substrate. Also, wasn’t urea used instead of ammonium as mentioned at a previous point? Later on, line 568-570, is this a typo “GDH1 catalyzes the final steps in the conversion of urea to glutamate and glutamine, which serve as the sources of cellular nitrogen”?

Good point, as the sentence was missing a part which explains that urea, which was indeed used as a nitrogen source in experiments with glucose-based media, is eventually converted to ammonia. Urea is deaminated using a bifunctional urea amidolyase, consequently converting urea into ammonia in two steps through an allophanate intermediate, unlike by urease (Linder 2019).

Lines 680-684 in the main text: modified “When the yeast had an abundant source of nitrogen, either by conversion of urea to ammonia (glucose condition) or by growth on ammonia itself, NADP-dependent glutamate dehydrogenase (GDH1) converted ammonia with the TCA cycle intermediate AKG into glutamate, which was then used for the amino acid biosynthesis.”

GDH is the only nitrogen fixation pathway with ammonium as nitrogen substrate. The part about alternative enzymes was referring to GLT and GDH2, which are involved when glutamate is the sole source of nitrogen. But this is indeed irrelevant here.

Line 689 in the main text: deleted “alternative enzymes of the nitrogen assimilation pathway”

Line 697 in the main text: deleted “GDH1 catalyzes the final steps in the conversion of urea to glutamate and glutamine, which serve as the sources of cellular nitrogen”

21. Page 30, line 649-653, the authors bring up a confusing correlation between carbon flux entering TCA cycle and malate-aspartate shuttle. Malate-aspartate shuttle net result is the transport of NADH from cytosol to mitochondria, which means carbon is not transported (as a net result). The authors need to elaborate, or this statement needs to be removed or rewritten for clarity.

We agree with the Reviewer that because of malate-aspartate shuttle, carbon is not transported (as a net result). Therefore, sentence is re-written to say that the shuttling and carbon transport are two separate things that happened at the same time during Nlim.

Lines 790-792 in the main text: modified “Our simulation results could not explain why Nlim phase, when more carbon entered the TCA cycle, was associated with the increase in internal cycling for NADH transport, also known as malate-aspartate NADH shuttle [41].”

22. Page 32, line 706-711, I see that the authors are discussing kcat values here. This statement needs to be placed at a point before mentioning modeling results so that readers can have correct interpretations of the results.

We agree with the Reviewer that statement about how k_cat_ values were chosen needs to be placed before describing modelling results. Therefore, 2 sentences have been removed from here. Only sentence referring to [48] remains to explain about the consequences of non-matched k_cat_ values.

Lines 859-861 in the main text: the following sentence remains “Enzyme-constrained metabolic models developed in this study used not fully matched k_cat_ values that can notably increase the prediction errors [48].”

Lines 860 in the main text: deleted “enzyme k_cat_ values from many different organisms, except *R. toruloides*, as such data are not available. When data are missing, k_cat_ values from similar substrates, reactions or other organisms are assumed.”

Is the kcat used the highest value among multiple values for multiple organisms? If this is the case, should kapp be preferred even if its value exceed kcat value?

Of course, if *in vivo* kapp exceeds kcat value it should be preferred, but any reliable *in vivo* data should be preferred if one aims to use a computational model to replicate *in vivo* conditions. Our calculated k_app_ values are based on model-predicted fluxes. In the ec-modelling framework, individual reactions are constrained by a combination of measured enzyme levels and k_cat_ values (for those reactions that we have such information of course), which do not allow for k_app_> k_cat_. This has been discussed in detail in our response to comment 13.

23. Were C/N ratios accounted for nitrogens in both ammonium and urea? On page 34, line 748-750, how exactly were the C/N ratios controlled? At what time points was C/N ratio control applied (e.g., end of exponential phase which is before N-lim phase)? Was C/N ratio value controlled by continuous or fed-batch mode?

We have specified that the C/N ratio was set at the beginning of the cultivation (t=0). Throughout the experiment, it was not controlled.

Lines 174-176 in the main text: modified “Cultures were grown at a starting molar C/N ratio of 69 (glucose/urea) and 80 (xylose- or acetate/ammonium sulfate), which will result in nitrogen limitation that is known to induce lipid accumulation [12].”

Lines 914-916 in the main text: modified “At the start of cultivation, the (C/N) molar ratio of the media in bioreactors was set to 69 (glucose/urea) and 80 (xylose- or acetate/(NH4)2SO4).”

24. Page 43, if no proteomic data is available, should the reactions not be constrained by enzyme level instead of using made-up enzyme levels? This can probably be done by setting a very high kcat (e.g., perhaps 1E18) in GECKO to make the constraint artificially inactive.

Reactions involving enzymes for which no proteomics data was available are not measured by made-up enzyme levels. If no enzyme level has been measured, this can be due to: (i) enzyme not being present, (ii) being present at too low concentration to be measured, (iii) being present but not being measured due to technical reasons related to protein extraction and/or mass-spectrometry. Indeed, one could argue that the enzyme constraint should disabled.

However, while we do not know the level of that individual enzyme, we do know that there is an upper bound on all enzymes together. In ec-models, enzymes for which no level has been determined (through whatever reason), are drawn from a "protein pool" pseudometabolite, whose quantity represents the total cellular protein content and how much of those proteins are enzymes. Not providing any proteomics data already drastically decreases the model's solution space, as already shown in the original GECKO paper (Sanchez et al. 2017, Mol. Syst. Biol.).

Now, in the hybrid situation where we have measurements for *some* enzymes but not for others, we can reduce the protein pool by the measured part. What remains is the non-measured part, which the model is now free to distribute to demanding reactions. As such, the model is constraint with measured protein levels, and for unmeasured proteins predicts their level. However, it will never predict a total enzyme usage that is higher than the amount of enzymes that are present in the cell. Artificially making the enzyme constraint inactive could result in flux distributions where the total enzyme usage is higher than the cell's biomass (although our parsimonious approach will of course already prevent this).

We are therefore confident that this approach improves prediction quality. However, on page 42 we aimed to signify for full disclosure that the original approach by which GECKO adjusts for the unmeasured protein was unable to handle the low protein levels we observed during Nlim. We therefore slightly adjusted this calculation, where we directly subtracted the measured enzyme concentration from the total enzyme concentration to obtain the unmeasured enzyme concentration, while the original approach instead readjusted the f-factor. The exact technical implementation is shown in the constrainEnzymes.m script."

Lines 1138-1142 in the main text: modified “An alternative approach to calculate the abundance of those enzymes for which no enzyme level had been measured was used as additional modification in addition to previously described modifications in the pipeline to handle the low protein levels observed in Nlim conditions.”

Lines 1142-1147 in the main text: modified “In this approach, we directly subtracted the measured enzyme concentration (Pmeasured) from the total enzyme concentration (enzymeConc) to obtain the unmeasured enzyme concentration (PpoolMass). Modifications to original approach, by which GECKO adjusts for the unmeasured enzyme concentration, are available from *generate_protModels* and *constrainEnzymes* functions at ‘customGECKO’ folder. These included ...”

Line 1162 in the main text: deleted “Non-measured part was pooled by subtracting Pmeasured (the measured mass of protein in the model) from enzymeConc.”

Minor concerns

1. Page 4, line 71, change “Origins of” to “Metabolic pathways producing”

Line 73 in the main text: modified “Metabolic pathways producing intracellular metabolite acetyl-CoA and a cofactor NADPH in *R. toruloides* have been the main focus of metabolic studies due to their central role in lipid biosynthesis.”

2. Page 5, line 101, change “enzymatic” to “biochemical”, as GEM also contains non-enzymatic transport, exchange, and spontaneous reactions.

Line 105 in the main text: changed “They contain all known biochemical reactions of the organism.”

3. Page 7, line 160-161, for better understanding, the authors need to rephrase the following: “induction of hydrolysate-tolerance and lipid accumulation genes without physiological changes regarding growth and substrate consumption”

Lines 164-174 in the main text: modified “*R. toruloides* CCT 7815 is a tolerant strain developed during a short-term adaptation of strain CCT 0783 [..] in sugarcane bagasse hemicellulosic hydrolyzate, demonstrating an increased lipid production without impacting growth and substrate consumption as a result of induction of hydrolysate-tolerance- and lipid accumulation-related genes [21].”

4. Page 8, line 161-162, “cultures were grown at a 162 molar C/N ratio from 69 to 80” should be rephrased to indicate C/N ratio being specific to provided substrate (as indicated in the Methods).

Lines 174-176 in the main text: modified “Cultures were grown at a starting molar C/N ratio of 69 (glucose/urea) and 80 (xylose- or acetate/ammonium sulfate), which will result in nitrogen limitation that is known to induce lipid accumulation [12].”

Lines 911-913 in the main text: modified “At the start of cultivation, the (C/N) molar ratio of the media in bioreactors was set to 69 (glucose/urea) and 80 (xylose- or acetate/(NH4)2SO4).”

7. In Figure 1’s footer, the authors need to explain the substrates denoted as “G”, “X”, and “A” (e.g., glucose is denoted as “G” ). It would be better if figure 1C is split into two separate bar charts for growth rates and glucose uptake rates.

Lines 187-189 in the main text: modified “Figure 1. Growth characterization on three carbon sources – glucose (G), xylose (X) and acetate (A) - during exponential growth (exp) and nitrogen limitation (Nlim) phases.”

Figure 1C has been split into two separate bar charts:

Lines 191-192 in the main text: modified “(C) Average specific growth rate, μ_average (h^-1^). (D) Substrate uptake rate, r_substrate (mmol/gDCW/h).”

8. The authors need to improve the English language usage in the manuscript for better text comprehension.

We have improved the use of English language throughout the manuscript:

Line 413 in the main text

Lines 522-523 in the main text

Lines 1142-1147in the main text

Lines 1206-1208 in the main text

# Reviewer #2:

# Minor concerns:

1. Please give the full name of “P/O ratio” when it appears at the first time.

Line 800 in the main text: gave full name of P/O ratio “As no experimental phosphate/oxygen (P/O) ratio has been yet reported in *R. toruloides*, it might be that the mitochondrial shuttling loop observed in our simulations in reality could correspond to a higher ATP requirement.”

2. Line 84, No XPK-related content was found in reference 10. Please double check.

Very well spotted. Zhu et al. 2012 indeed do not mention phosphoketolase (XPK) in the publication text, when reconstructingthe central carbon and lipid metabolism of *R. toruloides*. However, the protein coding gene of XPK can be found in their deposited genome of *R. toruloides* strain NP11 at NCBI database under the identifier XM_016418129.1.

Nevertheless, as the XPK-related content was not described in reference 10, we have added additional reference 14 “Evans CT, Ratledge C. Induction of xylulose-5-phosphate phosphoketolase in a variety of yeasts grown ond-xylose: the key to efficient xylose metabolism. Arch Microbiol. 1984;139: 48–52. doi:10.1007/BF00692711” that first demonstrated the presence of XPK in *R. toruloides*.

Lines 85-89 in the main text: inserted the second reference “The key differences included the synthesis of acetyl-CoA from citrate by ATP citrate lyase (ACL), synthesis of acetyl-CoA from xylulose 5-phosphate by phosphoketolase (XPK), and the conversion of S-malate into pyruvate by malic enzyme (ME) that provides for NADPH [10,14].”

3. Line 214-217, Is there any reference to support or explain the fact that the 68.5% of the carbon detected is due to cell aggregates? Why cell aggregates influence it?

Before sampling, optical density of cultures was determined via spectrophotometer. Biomass optical density data were calibrated by gravimetric cell mass measurements (lines 942-943 in the main text).

We have now referenced in the main text Haaber et al. 2016 and Koch 1970 to explain that optical density measurement is based on light scattering by the cells in the liquid and this is correlated to cell numbers under equal size and distribution assumption. If this asumption is not met due to aggregation, optical density measurements will underestimate the actual number of cells in the liquid.

There are series of research papers that provide evidence for underestimation of biomass concentration by optical density measurements in liquid cultures with cell aggregates. Haaber et al. 2016 (<https://doi.org/10.1016/j.mimet.2016.04.004>) showed that optical density measurements deliver slower growth of *S. aurus* in comparison with dry weight measurement in the same experiment.

Kragh et al. 2018 (<https://doi.org/10.1128/AEM.02264-17>) investigated ways to reduce aggregation in *P. aeruginosa* and emphasized the importance of homogeneity of liquid microbial batch cultures to increase reproducibility of experiments within and between laboratories.

Another study by Illarionov et al. 2021 (<https://doi.org/10.1128/AEM.03100-20>) reported aggregate formation that did not allow researchers to measure cell density in *R. toruloides* during growth on glucose-based synthetic medium.

To summarize, mass balance, expressed as a ratio of biomass, carbon dioxide and glucose (in C-mol), was disturbed in glucose condition due to microbial turbidity-based measurements of biomass concentration. Cell aggregate formation led to a lower measured absorbance values as compared to absorbance in a homogeneous liquid microbial culture.

Lines 277-282 in the main text: inserted “Mass balance calculation took into account glucose uptake, carbon dioxide production and biomass (in C-mol). As our biomass measurements were based on optical density, which relies on the assumption that cells are evenly distributed and of equal size [24,25], it may underestimated the actual cell concentration in liquid culture when aggregates are formed.”

4. Line 222, Please correct the spelling in the coordinate units of figure 1b and 1d. What is the color stands for in figure 1c, 1e and 1f. Please make it clear.

Figure 1B: coordinate unit changed from (g_lipid/gDW) to (g_lipid/gDCW);

Figure 1D (now 1E): coordinate unit changed from (g_protein/gDW) to (g_protein/gDCW);

The legend explaining color stands in figures 1c, 1e (now 1f) and 1f (now 1g) has been added to figure 1.

5. Many supplement figures don’t have a detailed figure legend. Please give more details. Readers should understand the figure based on its legend.

Lines 1438-1794 in the main text: supplement figure legends have been updated.

**Have the authors made all data and (if applicable) computational code underlying the findings in their manuscript fully available?**

### Reviewer #1: No: The github page mentioned in the manuscript is inaccessible <https://github.com/alinarekena/ecRhtoGEM>

We have made the repository public.

### Reviewer #2: Yes
